# In vivo assessment of collagen transdermal absorption in murine and human skin using photoacoustics microscopy

**DOI:** 10.1038/s41598-026-54420-4

**Published:** 2026-05-21

**Authors:** Jia Li, Zhongsheng Sun, Yahan Pang, Guangxian Zhang

**Affiliations:** 1 Guangzhou MUSE Biotechnology Co., LTD, Guangzhou, Guangdong China; 2 Department of Plastic and Reconstructive Surgery, Guangdong Second Provincial General Hospital, Guangzhou, Guangdong China; 3 Guangdong Country Garden School, Foshan, Guangdong China; 4 Department of Biochemistry and Molecular Biology, Guangzhou University of Chinese Medicine, Guangzhou, China

**Keywords:** Biological techniques, Medical research

## Abstract

The therapeutic potential of collagen for transdermal applications has been hindered by its limited penetration efficiency due to its high molecular weight. This study systematically evaluates the transdermal behavior of C3-modified collagen (ICG-C3) in murine, rat, and human skin using photoacoustic imaging (PAI). Dynamic PAI monitoring revealed that ICG-C3 exhibited enhanced penetration (3-fold higher dermal signal intensity than free ICG, *P* < 0.05) and prolonged retention (a 181.6% longer half-life than unmodified collagen, *P* < 0.001) in animal models. Human trials further demonstrated that the efficacy of ICG-C3 was dependent on the anatomical site: it achieved a penetration depth of 210 ± 30 μm (*P* < 0.01) in dorsal skin (characterized by a thick stratum corneum) and it exhibited a reduced signal decay rate to -25% of controls (*P* < 0.05) in ventral skin (with high sweat gland density). Non-destructive PAI visualized the directional migration of ICG-C3 along skin appendages. Our findings validate the cross-species universality of C3 modification and establish PAI as a robust tool for real-time transdermal analysis, offering critical insights for developing collagen-based therapeutics and advancing photoacoustic imaging in biomedical research.

## Introduction

Collagen is one of the most abundant structural proteins in the human body, widely distributed in tissues such as skin, bones, and cartilage, where it plays critical roles in supporting cellular architecture, maintaining skin elasticity, and facilitating tissue repair^1,2^. These functional attributes have driven significant interest in its therapeutic applications^3^. However, the transdermal delivery of collagen is severely limited by its high molecular weight, which hinders penetration through the skin barrier^4,5^. In recent years, diverse strategies—including chemical modifications, nanotechnology, and permeation enhancers—have been proposed to improve collagen’s transdermal permeability^6–8^. Despite promising in vitro results, these approaches face substantial challenges in practical clinical applications. First, the skin’s barrier function, governed by the tightly packed lipid bilayers of the stratum corneum, remains a formidable physical obstacle even with permeation enhancers^9^. Second, variable outcomes arise from individual differences in skin types and collagen’s inherent structural heterogeneity, leading to limited predictability in real-world settings.

Current research on transdermal collagen delivery remains exploratory^10^. Most studies focus on low-molecular-weight collagen fragments or degradation products, whereas investigations into the skin penetration efficiency of intact collagen are scarce^11^. Additionally, two critical challenges persist: (1) achieving efficient collagen penetration without compromising the skin barrier and (2) quantitatively monitoring the penetration process. Photoacoustic imaging (PAI), an emerging multimodal technology combining optical excitation and ultrasonic detection, offers high-resolution deep-tissue imaging capabilities. Unlike conventional optical microscopy, PAI provides enhanced contrast, resolution, and depth penetration, particularly for biological tissues rich in light-absorbing chromophores such as hemoglobin and melanin^12,13^. These advantages make it uniquely suited for dynamic analysis of drug penetration and deep-tissue structural characterization.

In this study, we employed PAI to investigate the transdermal behavior of collagen variants. An evaluation system was established using indocyanine green (ICG)-labeled collagen, with two experimental approaches: (1) animal model studies to assess skin penetration kinetics and half-life, and (2) human volunteer trials comparing collagen permeation across anatomical regions (ventral and dorsal forearm). To our knowledge, this is the first application of PAI for real-time, in vivo, comparative analysis of collagen penetration dynamics and metabolic profiles. Our findings not only validate the technical superiority of PAI in transdermal collagen research but also provide a robust framework for developing functional collagen-based therapeutics and transdermal delivery systems.

## Materials and methods

### Collagen preparation and labeling

C3 Collagen was purchased from Guangzhou Meishen Biotechnology Co., Ltd. (Cat. No. 250147) and was described as a permeable recombinant human collagen peptide with a molecular weight of approximately 20 kDa. To prevent impurities from interfering with the labeling reaction, the C3 collagen was purified and subjected to buffer exchange using an ultrafiltration tube (Millipore, Cat. No. UFC9010). It was first washed three times with 10 mM PBS, followed by three washes with a 10-fold volume of 0.1 M NaHCO3 (pH 8.3). The purified C3 collagen was subsequently concentrated to 10 mg/ml (measured using the M5 HiPer BCA Protein Assay Kit, Cat MF071-01, Mei5bio). Indocyanine green N-hydroxysuccinimide (ICG-NHS, Cat R-TE-157, Ruixibio) or FD1080-NHS (Cat R-NI-002, Ruixibio), dissolved in DMSO, was added, with the amount of NHS ester calculated according to the formula:

1 $$\begin{aligned} {\mathrm{NHS}}\_{\mathrm{ester}}\_{\text{weight }}\left[ {{\mathrm{mg}}} \right]{\text{ }} & = {\text{ 8 }} \times {\text{ amino}}\_{\mathrm{compound}}\_{\text{weight }}\left[ {{\mathrm{mg}}} \right] \\ & \quad \times {\text{ NHS}}\_{\mathrm{ester}}\_{\mathrm{molar}}\_{\text{weight }}\left[ {{\mathrm{Da}}} \right]{\mathrm{/amino}}\_{\mathrm{compound}}\_{\mathrm{molar}}\_{\text{weight }}\left[ {{\mathrm{Da}}} \right] \\ \end{aligned}$$

Indocyanine green (ICG) and FD1080 were selected as photoacoustic tracers due to their high optical absorption in the near-infrared (NIR) spectrum, which facilitates high-contrast imaging at significant tissue depths while minimizing autofluorescence. Both dyes were utilized in their N-hydroxysuccinimide (NHS) ester forms to enable stable covalent conjugation to the primary amines of the collagen molecules, ensuring that the tracked photoacoustic signals accurately represented the transdermal migration of the collagen-dye complex.

After vortex mixing, the mixture was incubated overnight at 4 °C in the dark for labeling. The labeled product was washed four times in the dark using 10 volumes of 10 mM PBS in an ultrafiltration tube to remove residual free NHS ester. The concentration of the labeled product was adjusted to 1 mg/ml with 10 mM PBS, filtered through a 0.22 μm filter, aliquoted, and stored for future use. Collagen protein was labeled following the same procedure as for C3. The covalent nature of the ICG-C3 conjugation was ensured by the specific reaction between the NHS-ester group of the dye and the primary amines of the collagen, followed by rigorous ultrafiltration to remove any non-conjugated molecules, as evidenced by the distinct metabolic profiles observed in subsequent imaging studies.

### Experimental animals and samples

Healthy BALB/c mice (6–8 weeks old, body weight 20 ± 3 g) and Sprague-–Dawley (SD) rats (6–8 weeks old, body weight 200 ± 10 g) were purchased from Beijing Vital River Laboratory Animal Technology Co. Ltd. All animals were adult females housed in a specific pathogen-free facility with ad libitum access to food and water under a 12-hour light-dark cycle. All animal experimental protocols were reviewed and approved by the Institutional Animal Care and Use Committee (IACUC) of Golden Wing (Suzhou) Medical Technology Co., Ltd. (Approval No. GW-IACUC-2024-RD002 V1.0). The study was conducted in strict accordance with the Guide for the Care and Use of Laboratory Animals (NIH Publication no. 85 − 23, revised 1996). Upon completion of the experiments, both mice and rats were euthanized by cervical dislocation following anesthesia with 1.25% tribromoethanol (0.2 mL/10 g body weight), in accordance with AVMA guidelines. Death was confirmed by cessation of respiration and heartbeat and absence of corneal reflex. All animal experiments were conducted in accordance with ARRIVE guidelines to ensure ethical and transparent reporting of in vivo research.

### Animal skin penetration PA imaging

The dorsal skin of mice and rats was selected as the imaging target. The following formulations were evaluated: FD-1080, FD-1080-labeled collagen, indocyanine green (ICG), and ICG-labeled collagen. Prior to imaging, the skin was depilated to remove hair and inspected for integrity (i.e., no abrasions or erythema). Mice were anesthetized with isoflurane and immobilized on a heated platform at room temperature. Skin penetration was dynamically monitored using an AR (acoustic-resolution) photoacoustic imaging system (AR-PAI) at multiple time points: baseline (pre-application) and 1, 2, 3, 4, 5, and 10 min post-application.

The AR photoacoustic-microscopy (AR-PAM) system’s specifications are as follows: (1) Optical source: Tunable pulsed OPO laser (SpitLight EVO S OPO-100, InnoLas, Munich, Germany); (2) Excitation wavelength: Tunable according to experimental requirements (optimal excitation wavelengths for ICG and FD1080 were used in this study); (3) Laser energy: Pulse energy can be adjusted based on imaging depth and safety requirements; (4) Imaging speed: Dependent on scanning step size and imaging range; point-by-point scanning acquisition was performed in this study; (5) Ultrasound frequency: Central frequency of 25 MHz (ultrasonic transducer model: V324-SU, Olympus IMS, Waltham, USA; fractional bandwidth: 14 MHz; N.A.: 0.25). In addition, the system is equipped with a precision 3D motorized scanning stage (PSA2000-11, Zolix, Beijing, China) for positioning the imaging probe, and a two-channel data acquisition (DAQ) card (CS1422, Gage Applied Technologies Inc., Lockport, USA) digitizing the photoacoustic pressure signals at a sampling rate of 200 MS/s.

### Human skin penetration study

The human skin penetration study was reviewed and approved by the Medical Ethics Committee of Guangzhou Dermatology Hospital (Approval No. gzsp202464) on July 15, 2024. The study protocol was evaluated through a formal meeting review, ensuring compliance with ethical standards and the protection of participant rights in accordance with the Declaration of Helsinki. The trail has been registered in the National Health Security Information Platform Medical Research Registration and Filing Information System (No. MR-44-25-048541, 7/7/2025). Participation in the study was voluntary and required the written consent of the participants, and enrolled six healthy Asian female volunteers aged 30–40 years with intact, tattoo-free forearm skin. Test formulations included phosphate-buffered saline (PBS), ICG, ICG-labeled standard collagen (ICG-P), and ICG-labeled experimental collagen (ICG-C3).

The ventral and dorsal regions of the forearm were designated as application sites. Photoacoustic signals were acquired at early penetration time points (baseline and 1, 2, 3, 4, 5, and 10 min) and for half-life assessment (baseline, 1, 5, 15, 30 min, and 1, 2, 3, 4, 5, 6 h).

### Photoacoustic imaging analysis

PAI data were processed using MATLAB (Version 9.9, MathWorks, Natick, MA, USA). Regions of interest (ROIs) were manually segmented from PA images based on signal distribution. Penetration depth and half-life of collagen were calculated by analyzing temporal changes in PA signal intensity across skin layers.

### Statistical analysis

All data were presented as the mean ± standard deviation. Statistical analysis and graph preparation were conducted using GraphPad Prism software (version 10.2.1). For animal model experiments involving a small sample size (*n* = 3), nonparametric tests (Mann-Whitney U test) were employed to compare differences between groups. For larger data sets meeting parametric assumptions, a two-tailed unpaired Student’s *t* test was utilized. Statistical significance was defined as a *P* value < 0.05.

## Results

### Penetration assessment in murine models

**Fig. 1:**
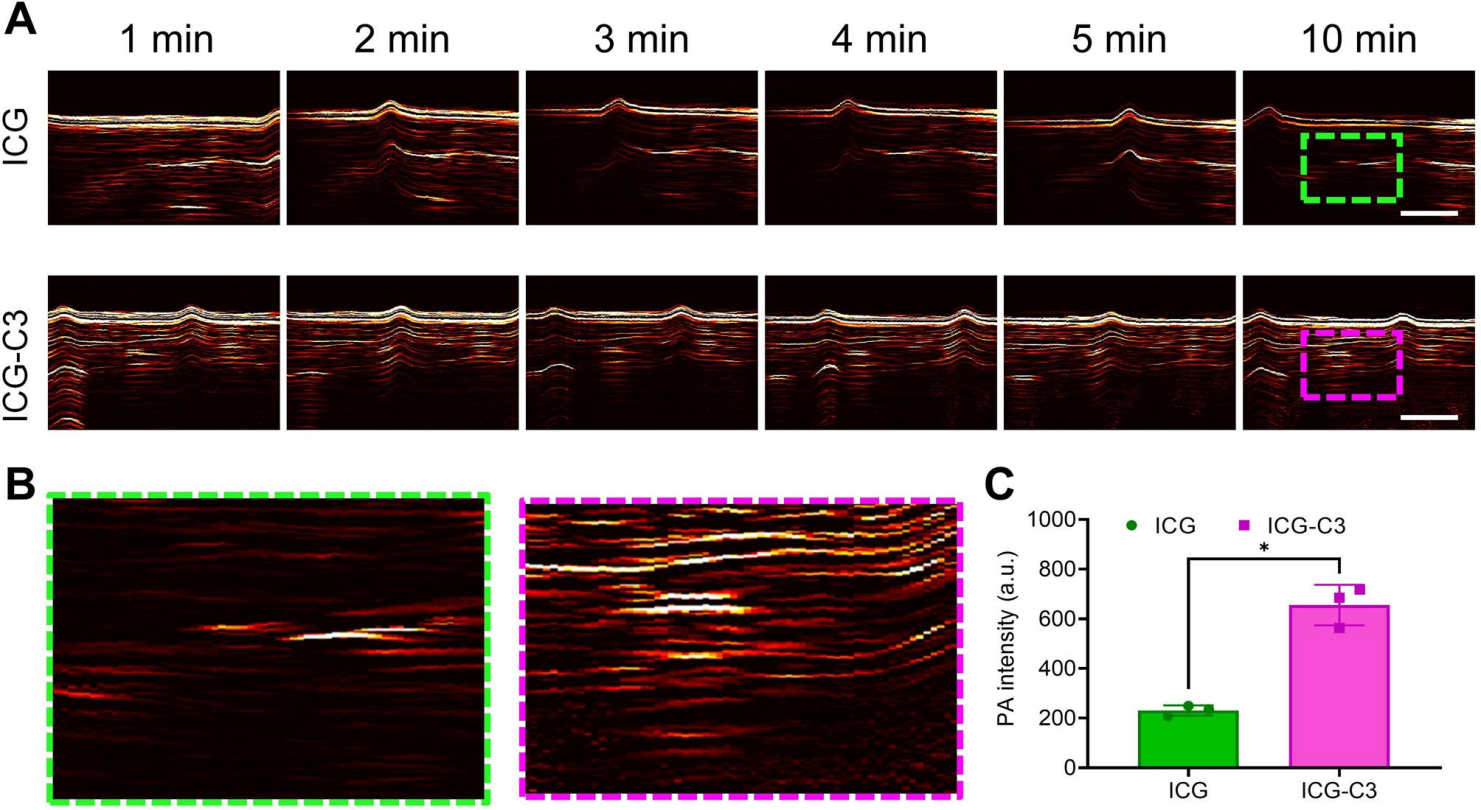
Evaluation of ICG and ICG-C3 skin penetration in mice. (**A**) PA imaging of the skin penetration of ICG and ICG-C3 in mice (Scale bar = 0.5 cm). (**B**) Enlarged PA images from (**A**). **C** Quantification of PA intensity from (**A**) (*n* = 3, *P* < 0.05).

We first compared the skin penetration dynamics of free ICG and ICG-labeled C3 collagen in a murine model. Photoacoustic image analysis revealed high-intensity epidermal signals in both groups at 1 min post-application (Fig. 1A). However, distinct temporal trends emerged: the ICG group exhibited rapid signal attenuation, with signals nearly undetectable by 10 min, whereas the ICG-collagen group demonstrated prolonged retention, maintaining strong photoacoustic signals at 10 min (Fig. 1B). Quantitative analysis further showed that the ICG-C3 complex enhanced dermal signal intensity by approximately 3-fold compared with free ICG at 10 min (*P* < 0.05, Fig. 1C), indicating that C3 collagen effectively penetrated into the dermal layer, with molecular weight differences significantly influencing transdermal efficiency.

### Penetration assessment in rat models

**Fig. 2:**
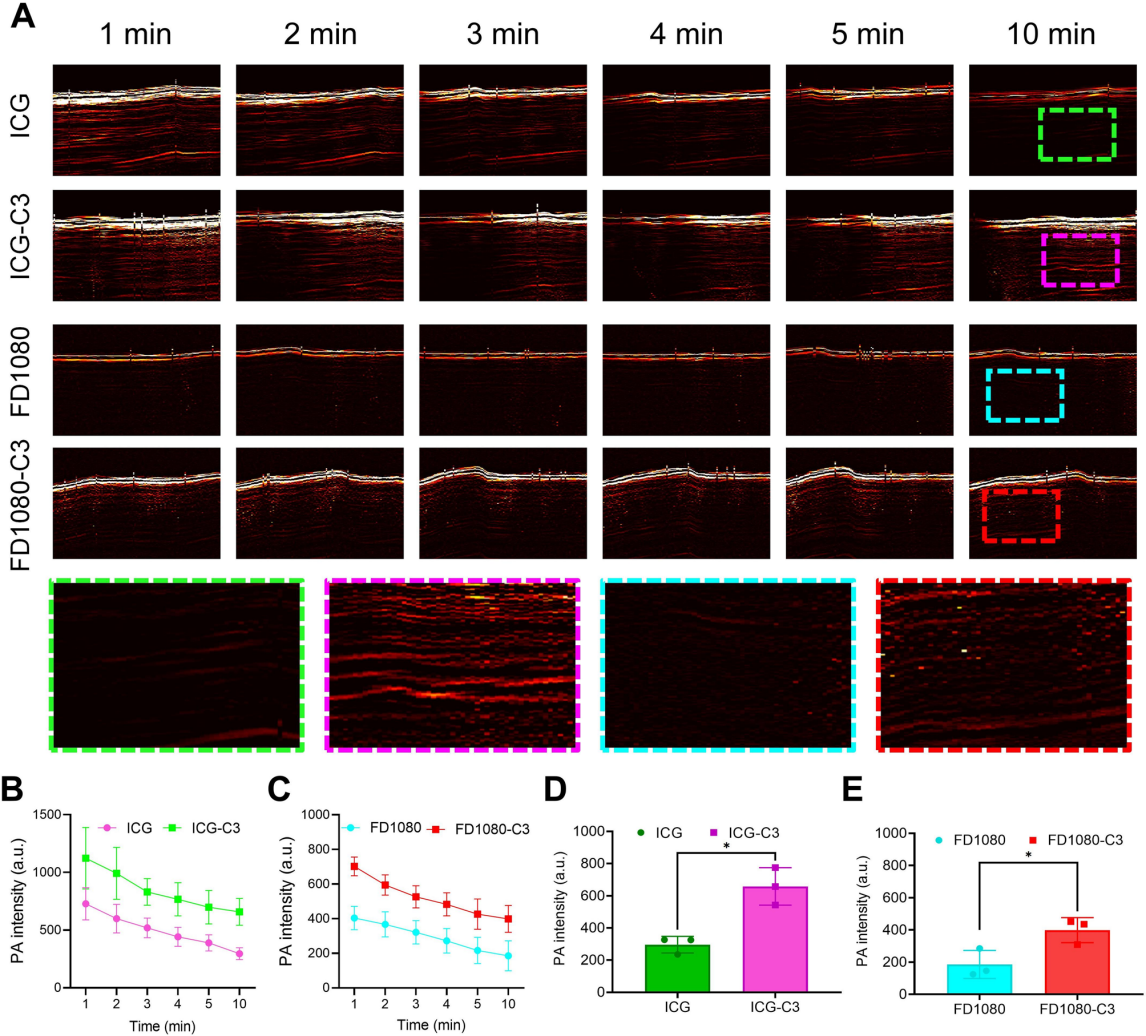
Evaluation of ICG/ICG-C3/FD1080/FD1080-C3 skin penetration in rats. (**A**) PA imaging of the skin penetration of ICG/ICG-C3/FD1080/FD1080-C3 in rats in different time point (Scale bar = 0.5 cm). (**B**) Temporal trends of PA imaging signals at different time points following topical application of ICG or ICG-C3. (**C**) Temporal trends of PA imaging signals at different time points following topical application of FD1080 or FD1080-C3. (**D**) Quantification of PA intensity at 10 min after application ICG or ICG-C3 (*n* = 3, *P* < 0.05). (**E**) Quantification of PA intensity at 10 min after application FD1080/FD1080-C3 (*n* = 3, *P* < 0.05).

**Fig. 3:**
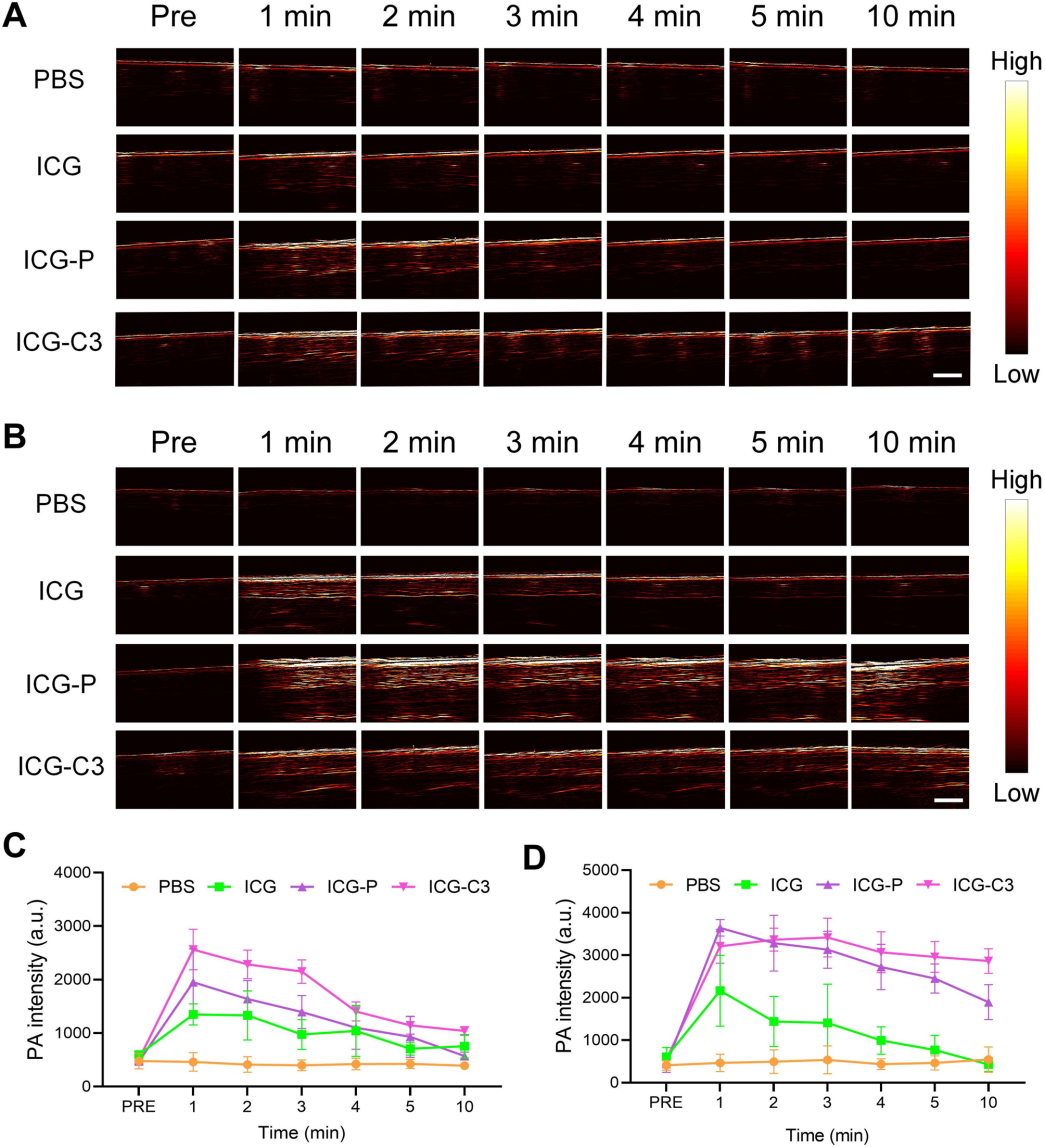
Evaluation of PBS/ICG/ICG-P/ICG-C3 on human skin penetration efficiency. (**A**) PA images of dorsal forearm skin at different time points following topical application of PBS, ICG, ICG-P, and ICG-C3 (Scale bar = 0.5 cm). (**B**) PA images of ventral forearm skin at different time points post-application of the test formulations (Scale bar = 0.5 cm). (**C**) Quantification of PA signal intensities corresponding to panel(**A**). (**D**) Quantification of PA signal intensities corresponding to panel (**B**).

To further investigate the transdermal penetration efficacy of C3 collagen, we systematically compared the permeation profiles of ICG, ICG-C3 complexes, FD1080, and FD1080-C3 complexes in a rat model (Fig. 3A). Photoacoustic imaging revealed initial epidermal signals in all four groups within 1 min post-application. Over time, free dyes (ICG and FD1080) exhibited rapid signal decay: ICG signals decreased to 53% of initial intensity at 5 min and became nearly undetectable by 10 min, while FD1080 showed similar attenuation trends. In stark contrast, collagen-conjugated groups (ICG-C3 and FD1080-C3) demonstrated enhanced penetration and prolonged retention (Fig. 2B, C).

A cross-group comparison at 10 min further validated the superior penetration efficacy of C3 collagen. The dermal signal intensities of ICG-C3 and FD1080-C3 were 2.22-fold and 2.84-fold higher than their free counterparts, respectively (*P* < 0.05, Fig. 3D, E), suggesting that C3 collagen modification significantly enhances the potential for deep dermal penetration.

### Penetration assessment in human volunteers

Building on the rodent model findings demonstrating enhanced penetration and prolonged retention of C3 collagen, we further validated its efficacy in the physiologically complex human skin system through PAI. Dynamic monitoring of ICG-C3 complexes versus ICG-labeled standard collagen (ICG-P) was performed on both dorsal (thicker skin, stronger barrier function) and ventral (thinner skin, higher sweat gland density) forearm regions in six healthy volunteers.

In dorsal forearm skin, ICG-C3 exhibited rapid penetration kinetics (Fig. 3A). At 1 min post-application, the dermal signal intensity of ICG-C3 peaked at 1.31-fold higher than that of ICG-P. Furthermore, ICG-C3 achieved a greater penetration depth compared with ICG-P (Fig. 3C).

The superior efficacy of C3 collagen was even more pronounced in ventral forearm skin (Fig. 3B). By 10 min, the dermal signal intensity of ICG-C3 was 1.82-fold greater than that of ICG-P. Notably, while free ICG and ICG-P groups showed a decreasing trend over time in ventral skin, the ICG-C3 group maintained relatively constant signals throughout the 10-minute observation window (Fig. 3D), suggesting its potential to prolong retention in the dermal layer by mitigating rapid clearance mechanisms.

### Quantitative assessment of transdermal retention

To evaluate the transdermal retention enhancement mediated by C3 collagen, we analyzed the metabolic decay profiles of ICG-P and ICG-C3 in both dorsal and ventral forearm skin.

**Fig. 4:**
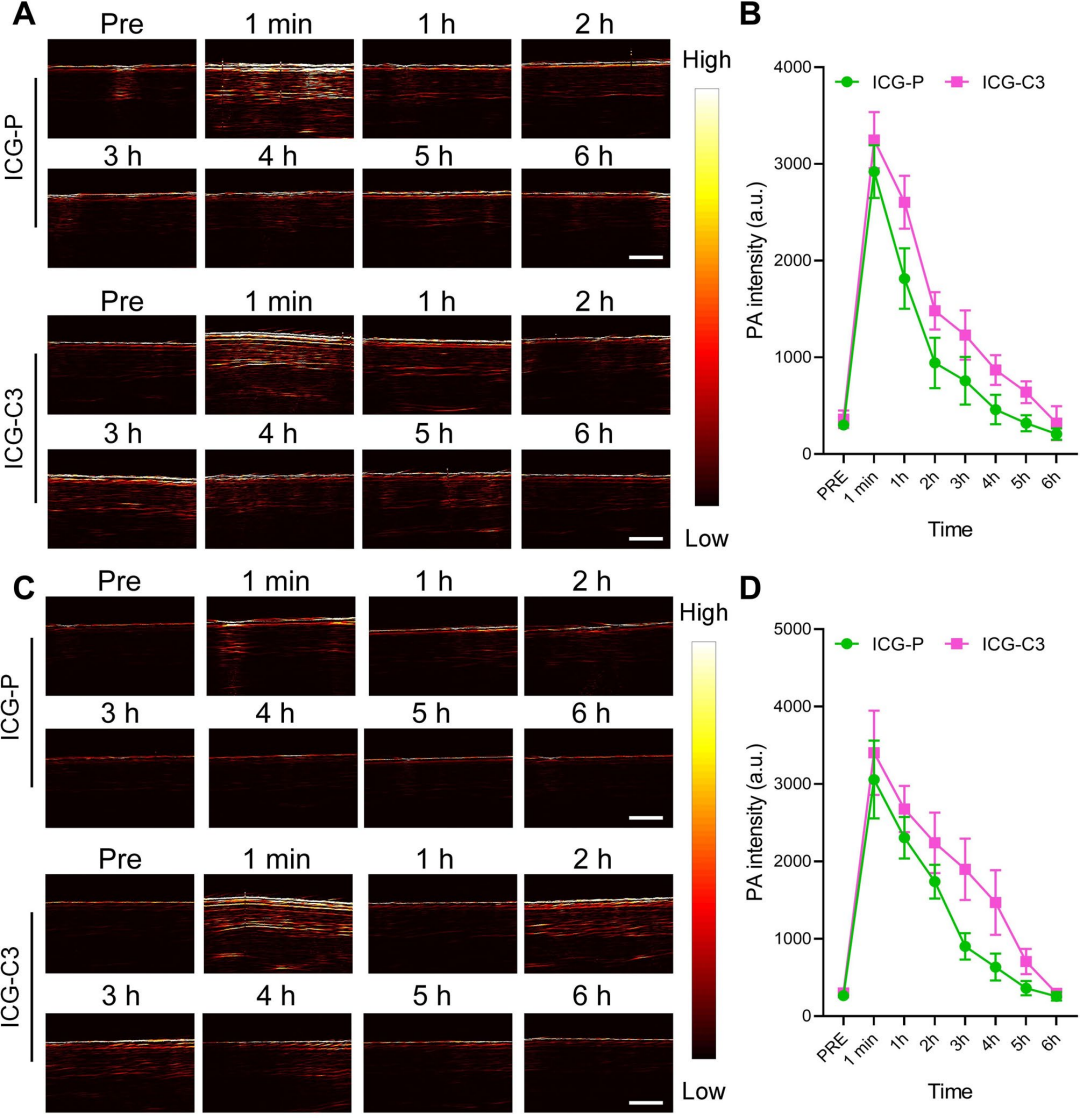
Half-life evaluation of ICG-P/ICG-C3 following topical application on human skin. (**A**) PA images of dorsal forearm skin at various time points over 6 h post-application of ICG-P/ICG-C3 (Scale bar = 0.5 cm). (**B**) Quantification of PA intensity of (**A**). (**C**) PA images of ventral forearm skin at various time points over 6 h post-application of ICG-P/ICG-C3 (Scale bar = 0.5 cm). (**D**) Quantification of PA intensity of (**C**).

In dorsal skin, the ICG-C3 group demonstrated prolonged retention characteristics (Fig. 4A, C). At 1 h post-application, the signal intensity of ICG-C3 (2603.64 a.u.) was 1.43-fold higher than that of ICG-P (1815.17 a.u.). Furthermore, in ventral skin, ICG-C3 exhibited consistently higher PA signals throughout the 6-hour observation period compared to ICG-P (Fig. 4B, D), confirming its superior retention efficacy in diverse anatomical regions.

## Discussion

This study evaluated the transdermal penetration and retention characteristics of C3-modified collagen in multiple models (murine, rat, and human skin) using PAI, which revealed its significant advantages over unmodified collagen.

In animal models, the ICG-C3 complex demonstrated markedly enhanced penetration efficacy, characterized by significantly higher signal intensity and prolonged retention compared with free ICG and unmodified collagen. This may be attributed to C3 collagen’s ability to retain the C-terminal nonhelical region of mature human collagen and undergo random mutation screening to identify transmembrane-penetrating mutants, thereby further influencing penetration efficiency. In human trials, the superior penetration efficacy of C3 collagen exhibited anatomical site-dependency. Regardless of whether it was the thicker stratum corneum and stronger barrier function in dorsal forearm skin or the higher sweat gland density in ventral forearm skin, ICG-C3 consistently showed prolonged retention rates in the dermal layer.

The cross-species efficacy of C3 collagen (murine, rat, and human) provides critical evidence for its clinical application. By extending collagen retention time in the dermal layer, C3 modification holds promise for enhancing therapeutic efficacy in skin repair, anti-aging, and localized drug delivery. Additionally, the dynamic monitoring capability of PAI offers a real-time evaluation tool for optimizing transdermal formulations, overcoming the limitations of destructive traditional histological methods. The characteristic stratified signal distribution observed in our PA images is a result of the high axial resolution of the AR system, which captures the differential optical absorption across the stratified layers of the skin (stratum corneum, epidermis, and dermis). Rather than the continuous uniform distribution seen in wide-field imaging, this pattern reflects the depth-resolved migration of the collagen as it traverses distinct biological interfaces.

The dynamic monitoring capability of PAI not only enables real-time visualization of transdermal processes but also provides multidimensional, quantitative parameters (e.g., penetration depth, signal decay rate, and spatial distribution patterns), offering novel insights for optimizing the pharmacokinetics of collagen in skin^14^. The use of NIR-absorbing tags such as ICG (~ 780–800 nm (NIR-I)) and FD1080 (~ 1060–1080 nm (NIR-II)) is critical for photoacoustic applications, as these wavelengths fall within the biological ‘optical window’ where light scattering and absorption by water and hemoglobin are minimized, allowing for the deep-tissue characterization of collagen penetration kinetics. Compared to traditional histochemical staining, PAI non-destructively captures penetration trajectories without disrupting the skin barrier, significantly improving experimental reproducibility and bridging preclinical-clinical data translation. Therefore, employing PAI to study collagen transdermal penetration allows comprehensive and systematic analysis of its dynamic distribution and metabolic processes in skin, providing reliable technical support for the development and optimization of collagen-based products^15^.

This study has several limitations. First, the limited sample size in human trials necessitates expanded cohorts to validate the impact of individual variability. Second, the precise chemical mechanisms underlying C3 modification remain unclear and require further elucidation through molecular dynamics simulations or in vitro binding assays. Finally, long-term safety profiles (e.g., skin barrier integrity, immune responses) demand systematic evaluation. Future research could explore synergistic effects between C3 collagen and other permeation enhancers or develop multimodal imaging strategies to track its metabolic fate.

## Conclusion

In summary, this study evaluated the transdermal permeability of collagen in vivo using PAI. Through investigating the penetration dynamics and half-life of ICG-labeled collagen, we demonstrated its superior skin penetration capability. These findings provide novel insights for the further development of collagen-based therapeutic and cosmetic products.

## Data Availability

All data generated or analyzed during this study are available within the Article.

## References

[1] Wang, S. et al. Promoting collagen synthesis: A viable strategy to combat skin ageing. *J. Enzyme Inhib. Med. Chem.* **40**, 2488821 (2025). 40213810 10.1080/14756366.2025.2488821 PMC11995770

[2] Danessa, G., Notario, D. & Regina, R. Effects of collagen-based supplements on skin’s hydration and elasticity: A systematic review and meta-analysis. *Indian J. Dermatol. Venereol. Leprol.* **91**, 1–11 (2025). 10.25259/IJDVL_1165_2023 40826844

[3] Pintea, A. et al. Peptides: Emerging candidates for the prevention and treatment of skin senescence: A review. *Biomolecules,* **15**, 88 (2025). 10.3390/biom15010088 PMC11762834 39858482

[4] Shi, S. et al. A highly biocompatible and bioactive transdermal nano collagen for enhanced healing of UV-damaged skin. *Int. J. Biol. Macromol.* **272**, 132857 (2024). 38834124 10.1016/j.ijbiomac.2024.132857

[5] Szumała, P., Jungnickel, C., Kozłowska-Tylingo, K., Jacyna, B. & Cal, K. Transdermal transport of collagen and hyaluronic acid using water in oil microemulsion. *Int. J. Pharm.* **572**, 118738 (2019). 31705977 10.1016/j.ijpharm.2019.118738

[6] Mortazavi, S. M., Mohammadi, V. S. & Moghimi, H. R. Topically applied GHK as an anti-wrinkle peptide: Advantages, problems and prospective. *Bioimpacts* **15**, 30071 (2025). 39963574 10.34172/bi.30071 PMC11830136

[7] Bhat, B. B., Kamath, P. P., Chatterjee, S., Bhattacherjee, R. & Nayak, U. Y. Recent updates on nanocosmeceutical skin care and anti-aging products. *Curr. Pharm. Des.* **28**, 1258–1271 (2022). 35319358 10.2174/1381612828666220321142140

[8] Li, M. et al. Preparation, characterization and ex vivo skin permeability evaluation of type I collagen-loaded liposomes. *Int. J. Nanomed.* **18**, 1853–1871 (2023). 10.2147/IJN.S404494 PMC10086223 37057190

[9] Sahle, F. F., Gebre-Mariam, T., Dobner, B., Wohlrab, J. & Neubert, R. H. Skin diseases associated with the depletion of stratum corneum lipids and stratum corneum lipid substitution therapy. *Skin. Pharmacol. Physiol.* **28**, 42–55 (2015). 25196193 10.1159/000360009

[10] Mizuguchi, T. et al. Three-dimensional analysis of water dynamics in human skin by stimulated raman scattering. *J. Phys. Chem. B*. **127**, 4952–4958 (2023). 37224384 10.1021/acs.jpcb.3c00103

[11] Tao, K., Zhu, H. & Wei, J. Anti-aging effect of low molecular weight recombinant humanized collagen on photo-aging by activating adherence junction signaling pathways. *Plos One*. **20**, e329460 (2025). 10.1371/journal.pone.0329460 PMC12396721 40880343

[12] Lin, L. & Wang, L. V. The emerging role of photoacoustic imaging in clinical oncology. *Nat. Rev. Clin. Oncol.* **19**, 365–384 (2022). 35322236 10.1038/s41571-022-00615-3

[13] Wang, L. V. & Hu, S. Photoacoustic tomography: In vivo imaging from organelles to organs. *Science* **335**, 1458–1462 (2012). 22442475 10.1126/science.1216210 PMC3322413

[14] Wang, L. V. & Yao, J. A practical guide to photoacoustic tomography in the life sciences. *Nat. Methods*. **13**, 627–638 (2016). 27467726 10.1038/nmeth.3925 PMC4980387

[15] Jadach, B., Mielcarek, Z. & Osmałek, T. Use of collagen in cosmetic products. *Curr. Issues Mol. Biol.* **46**, 2043–2070 (2024). 38534748 10.3390/cimb46030132 PMC10968853

